# Engagement science: The core of dissemination, implementation, and translational research science

**DOI:** 10.1017/cts.2020.8

**Published:** 2020-01-20

**Authors:** Paul Meissner, Linda B. Cottler, Milton “Mickey” Eder, J. Lloyd Michener

**Affiliations:** 1 Montefiore Medical Center, Albert Einstein College of Medicine, Bronx, NY, USA; 2 Clinical and Translational Science Institute, University of Florida, Gainesville, FL, USA; 3 Department of Family Medicine and Community Health, Clinical and Translational Science Institute, University of Minnesota, Minneapolis, MN, USA; 4 Clinical and Translational Science Institute, Duke University School of Medicine, Durham, NC, USA

**Keywords:** Engagement science, stakeholder engagement, community engagement, dissemination and implementation science, CTSAs

## Abstract

Stakeholder engagement is acknowledged as central to dissemination and implementation (D&I) of research that generates and answers new clinical and health service research questions. There is both benefit and risk in conducting stakeholder engagement. Done wrong, it can damage trust and adversely impact study results, outcomes, and reputations. Done correctly with sensitivity, inclusion, and respect, it can significantly facilitate improvements in research prioritization, communication, design, recruitment strategies, and ultimately provide results useful to improve population and individual health. There is a recognized science of stakeholder engagement, but a general lack of knowledge that matches its strategies and approaches to particular populations of interest based on history and characteristics. This article reviews stakeholder engagement, provides several examples of its application across the range of translational research, and recommends that Clinical Translational Science Awards, with their unique geographical, systems, and historical characteristics, actively participate in deepening our understanding of stakeholder engagement science and methods within implementation and dissemination research. These recommendations include (a) development of an inventory of successful stakeholder engagement strategies; (b) coordination and intentionally testing a variety of stakeholder engagement strategies; (c) tool kit development; and (d) identification of fundamental motivators and logic models for stakeholder engagement to help align stakeholders and researchers.

## Introduction

Translational scientists have increasingly advocated for team science, which offers institutions one way to support engagement in interdisciplinary dialogues that generate and answer new clinical and health services research questions [1]. One recent translational science manuscript points to engagement as a common element across the dissemination, implementation, and translational research domains and proposes the Integrative Framework of Dissemination, Implementation, and Translation (IFDIT), which places stakeholder engagement at its center. Articles about community engagement, among the most cited *Journal of Clinical and Translational Science* articles [2-6], often view communities as being represented by individual stakeholders or organizations. A current translational science challenge involves diversifying engagement across the spectrum of research. In addition to focusing on the types of individuals enrolled in a trial, translational science seeks to develop teams by engaging types of stakeholders who represent research administrative, public health, philanthropic, and corporate interests. One stakeholder engagement taxonomy identifies seven types of stakeholders: patients, the public, providers, purchasers, payers, policy makers, product makers, and principal investigators [7]. Characteristics of engagement in team science include bidirectionality, collaboration, reciprocity, synergy, transparency, and trust [8,9]. These characteristics are described in comparative effectiveness (CER) [10], patient-centered outcome (PCOR), community-based participatory (CBPR) [11], and practice-based research network (PBRN) literatures [12]. Engaging diverse voices and perspectives is credited with increased improvements in communication, research design, and recruitment strategies. In distinguishing between a team and its activities or between engagement and research strategies, inclusion of external voices and perspectives on research teams is credited with fostering collaboration, partnerships, and coalitions by addressing power, infrastructure, and resource imbalances [9]. The contributions of external research team members are described most often in accounts of prioritizing, developing, and conducting specific research projects and less frequently in reports on dissemination and implementation research [9,10,13].

Leppin et al. [1]point out that engagement is not formally described in the context of early phase research. Yet, when queried, researchers conducting basic and preclinical research recognize they practice team science and routinely conduct engagement activities with scientific, technical, and industry colleagues. Early stage research is characterized by broad pursuit of exploratory scientific and technical concepts among a relatively limited pool of investigators. Such specialized investigators often know each other and have professional forums (publications, conferences) in which to share progress and problems. While these exploratory activities often do not yield avenues worthy of future pursuit, they provide their participants with extensive practice in one form of engagement.

The Partners for the Advancement of Community Engaged Research (PACER) group is one such place to share progress and problems. PACER was established in the first round of Clinical Translational Science Awards (CTSAs), to supplement the formal CTSA structure, in which only one person per institution is allowed to be a voting member. PACER is an open group, in which multiple interested members representing various stakeholder constituencies (e.g., academic, research, practice, and community) can participate, with an aim to improve public health through building community–academic research partnerships. PACER meetings provide dissemination opportunities about community engaged methods research, metrics, and evaluation; they foster collaboration through networking and sharing among individuals and groups engaged in clinical and translational activities.

## Discussion

To best illustrate the role and impact of stakeholder engagement in the entire range of translational research, we identified examples of stakeholder interests creating bridges across the translational continuum from basic science to clinical care. We further recognize the contributions of an expanded infrastructure for team science to succeed (e.g., incorporation of communication, epidemiological, and mapping tools in public health programming).

Beginning with a rare disease, the value and methods of systematic patient and stakeholder engagement have been pioneered and demonstrated by the cystic fibrosis (CF) community for more than a decade. Key to the dramatic improvement in survival of children with CF from the 1960s, when most children with CF did not reach school age, to the median age of survival now in the forties, has been a strong patient community, which developed a strategic plan, targeted funding, and advocated and invested in a range of research reflective of the translational continuum, from basic science to quality improvement. Central to all of these has been a focus on engaging people with CF and their families, including in clinical practice guideline committees and data collection methods. Methods of patient engagement in a research priority setting have particularly advanced – despite challenges of patients being widely distributed and not able to meet easily due to infection risk – including sequential use of online surveys promoted through social media to set research priorities [14]. While no single set of best practices emerged, clear value has been found both in deep engagement of a small number of trained stakeholders in research and in wide, “crowd-sourced” online engagement [15].

A classic example of community engagement in dissemination and implementation is town hall meetings linked to stakeholder action. The University of Florida (UF) CTSA hub hosts a bimonthly town hall meeting called “Our Community, Our Health (OCOH),” where investigators share the latest research findings about a topic and community members share issues relevant to the disorder/illness from a personal perspective. The one-hour session is streamed live; questions are submitted in real time for the panelists. Using the OCOH structure, the UF hub polled its Community Advisory Board (CAB) about top health concerns in their areas in Florida. Addiction (primarily opioid use) was a major problem deserving attention, which prompted UF’s Community Engagement program to reserve one OCOH for this topic. The session was well received; in fact, it was so successful that the CAB requested a second OCOH on this topic and a third on Medical Marijuana, which featured the Health Science Administrator from the National Institute on Drug Abuse. While any university or institution may suggest a topic of interest in their population and suggest individuals to participate, the UF hosts each program and web-streams it. In this way, new findings and local perspectives are disseminated widely to multiple communities both nationally and internationally. The UF further encourages the local community to use this opportunity to meet in conjunction with OCOH sessions to inform researchers of topics and solutions in which they are interested.

As a result of these three addiction-focused town hall meetings, new ideas for opioid-focused and drug use research emerged, including distribution of deactivation pouches for expired and unused opioids, increased distribution of naloxone by Community Health Workers, and the impact of adoption of a neighborhood health approach to both the opioid epidemic and medical marijuana. The forums through the facilitation of consensus among the different sectoral participants confirmed that person-centered engagement was valued and accepted as the norm to help reduce drug use. Participants acknowledged that it was useful to hear how other communities were handling their drug use issues. Regarding OCOH metrics, software allowed tracking Twitter impressions, calls, texts; the questions tweeted are available for later use in assessing the reach and value of message dissemination.

A third, broad example of engagement and stakeholder action focuses on reduction in population-based risk factors for common diseases. Widespread variation in mortality rates in communities across the USA have been highlighted by the Robert Wood Johnson Foundation and provided online at the county level at https://www.countyhealthrankings.org/; census-track-level data on risk factors for the top 500 cities is now available from the Centers for Disease Control and Prevention at https://www.cdc.gov/500cities/. In response to public access to reliable data, hundreds of local community coalitions have formed to improve local health outcomes (https://www.practicalplaybook.org/page/about-find-partnership), leading in turn to community-led implementation projects (https://buildhealthchallenge.org/), which aim to improve local health outcomes, sometimes with impressive results [16]. These efforts to provide community and stakeholder access to reliable health data have recognized the need for the continued development of translational science expertise. This includes accommodating new implementation and dissemination science expertise, buttressing activities to share nuanced understandings, and foster capacity to further the application of ideas and practices successfully within local settings. These collaborations further recognize that a response to data-informed, community-generated initiatives may require strategic collaboration decisions on how to best use and leverage finite research resources.

Given the varied and often powerful examples of stakeholder engagement across the translational research continuum, CTSAs, with their unique geographical, systems, and historical characteristics, have the potential of deepening our understanding of engagement science and methods within implementation and dissemination research. This can contribute to our understanding of contexts and conditions within which partnerships function across the research continuum. CTSAs can champion the use, assessment, and further refinement of existing tools to advance community–academic and team science partnerships and engagement strategies [15]. Using implementation and dissemination science can help answer fundamental questions regarding what partnership characteristics match to successful collaborations and to successful action within particular populations of interest. The study of engagement, particularly with a focus on community, will help to explore and transform the conditions within which research occurs between basic science disciplines as well as outside academic science centers from the bench to the community.

## Recommendations

These examples and observations lead to several recommendations that could result in identifying a set of engagement practices that advance translational research. Concomitantly, they point to opportunities for further studying the risks and benefits of incorporating diverse community and stakeholder interests into implementation and dissemination research.

First, the examples highlight that CTSAs, to fully engage in translational research, have an obligation to develop an inventory of successful stakeholder engagement strategies. The CF and OCOH examples show that this can be accomplished through engagement with patients and advocates as well as surveys in the field. Team science can encourage exploration of engagement practices utilized by colleagues from other disciplines such as marketing, social science, and education, broadening the search for promising research approaches, methods, and strategies.

Second, the case studies presented here are constituted only as a convenience sample. Many CTSAs have developed valuable programmatic activities which would be uncovered through the inventory process. At the same time, engagement with a broader investigator community would allow the identification of approaches not already considered. This would offer CTSAs the opportunity to collaborate and coordinate to intentionally test a variety of those stakeholder engagement strategies and identify those which accelerate the process of developing results that make a difference in health and that reduce health disparities.

Third, similarly the inventory and interprofessional exploration should stimulate CTSAs to move beyond general engagement strategies to develop a tool kit for engagement that might be considered (or avoided) in different stakeholder contexts, audiences, and objectives. A common theme of PACER meetings has been the engagement role of CTSA hubs as conveners of local and state stakeholders, who can help share and coordinate data, analyze plans, coordinate and assess interventions, and disseminate promising tailored practices.

Finally, our fourth recommendation is that CTSAs should work collaboratively. They must approach team science as an opportunity to further the science of stakeholder engagement, identify fundamental influencers with logic models for stakeholder engagement that will help align stakeholders and researchers, and speed up the incorporation of research findings into practice, and improved health. Alternately, this might take the form of baby steps around supporting integrating engagement into translational science metrics for assessing how choices among multiple potential stakeholder responses create community benefit.

We know that CTSAs can play an important role in accelerating translational research through stakeholder engagement. It is time we learned how and why engagement matters, so that the scientific basis of stakeholder (and community) engagement is truly embedded at the core of the dissemination and implementation science framework.
